# Primary laryngeal paraganglioma with multisystem metastases: A case report

**DOI:** 10.1097/MD.0000000000050620

**Published:** 2026-09-18

**Authors:** Xia Xu, Shuting Ma, Yaping Zou

**Affiliations:** a Department of Pathology, Affiliated Jinhua Hospital, Zhejiang University School of Medicine, Jinhua, Zhejiang Province, China; b Department of Otorhinolaryngology, Head and Neck, Jinhua Maternal and Child Health Hospital, Jinhua, Zhejiang Province, China; c Department of Otorhinolaryngology, Head and Neck, Affiliated Jinhua Hospital, Zhejiang University School of Medicine, Jinhua, Zhejiang Province, China.

**Keywords:** laryngeal paraganglioma, laryngeal tumors, neuroendocrine neoplasms

## Abstract

**Rationale:**

Laryngeal paraganglioma (LP) is a rare neuroendocrine tumor with limited evidence on its metastatic behavior. This case is unique because it describes the diagnostic process, therapeutic decisions, and clinical outcome of LP with multisystem metastases, contributing to a better understanding of its aggressive course and management challenges.

**Patient concerns:**

The patient presented with progressive dysphagia, which was later accompanied by hoarseness and dyspnea.

**Diagnoses:**

Clinical examination and imaging revealed a mass involving the larynx. Histopathological and immunohistochemical analyses confirmed LP.

**Interventions:**

The patient underwent tracheostomy after declining partial laryngectomy. Due to tumor progression 5 months post-surgery and dysphagia, partial laryngectomy with extensive tumor resection was performed.

**Outcomes:**

Four months postoperatively, recovery was uneventful, with no local recurrence and successful tracheostomy tube removal. However, at 13 months after initial diagnosis (November 22, 2020), multiple systemic metastases were detected in the lungs, pancreas, and subcutaneous tissues. The patient succumbed to systemic failure on February 9, 2021.

**Lessons:**

LP diagnosis relies on combined histopathological and immunohistochemical confirmation. Complete surgical resection is critical for preventing recurrence. Once metastasis develops, the prognosis remains poor, underscoring the importance of early diagnosis and multidisciplinary treatment.

## 1. Introduction

Laryngeal paragangliomas (LPs) are rare neuroendocrine tumors primarily arising from the superior laryngeal paraganglia.^[1]^ LPs are unique among laryngeal neuroendocrine neoplasms for their female predominance (male-to-female ratio ~1:3).^[2,3]^ Although most LPs follow a benign clinical course, they possess malignant potential, with metastatic disease present at diagnosis in over 10% of paragangliomas, highlighting the critical need for comprehensive initial staging.^[4,5]^ Diagnosis relies on histopathological identification of the characteristic Zellballen pattern, confirmed by immunohistochemical positivity for synaptophysin (Syn), chromogranin A (CgA), and S-100 in sustentacular cells. Curative treatment requires complete surgical resection with clear margins (R0). Nonetheless, malignant or metastatic LPs are associated with poor prognosis and present substantial therapeutic challenges, as illustrated in previous reports. This report describes an aggressive case of primary LP in an older adult, culminating in multisystem metastases and death on February 9, 2021, thereby contributing to the sparse literature on this rare and aggressive variant.

## 2. Case report

### 2.1. Patient demographics and presentation

A 72-year-old male patient presented in October 2019 with progressive dysphagia (Visual Analogue Scale 6/10, indicating moderate dysphagia) and inspiratory stridor (grade II, per British Thoracic Society criteria).

### 2.2. Clinical findings

**Figure 1.**
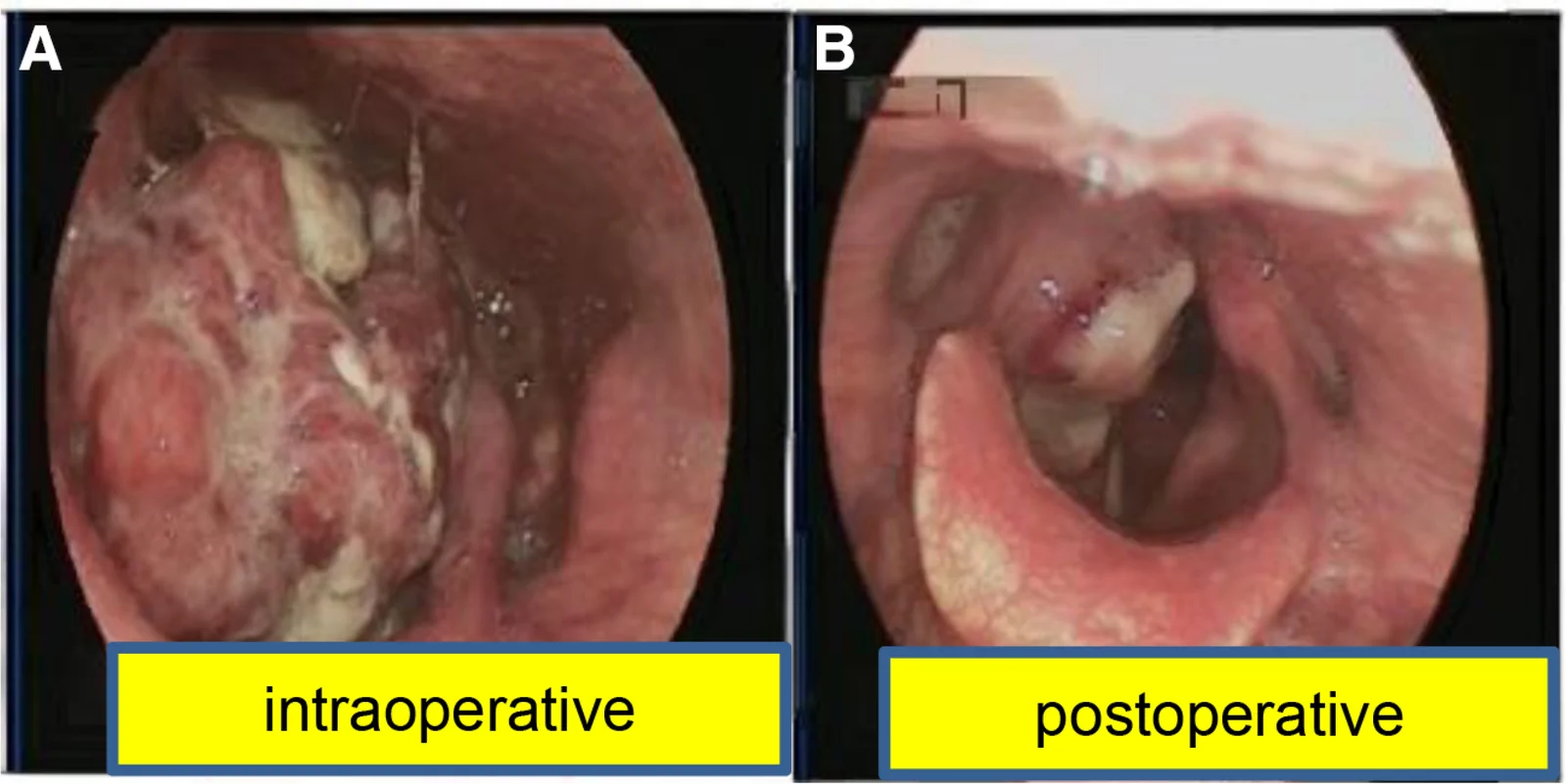
Before surgery, huge masses were found to block the entrance of the larynx. Intraoperative biopsy revealed that the masses were brittle in texture and easy to bleed (A). Postoperative review of laryngoscopy revealed that the base of the masses was located at the back end of the right ventricular band, and the right vocal cord had poor mobility (B).

Laryngoscopic evaluation revealed a 5 × 5 cm ulcerated supraglottic mass with purulent exudate, obliterating the right pyriform sinus (Fig. 1). Functional assessment demonstrated a Voice Handicap Index–10 score of 28/40 (indicating moderate voice handicap) and a maximum phonation time of 6 seconds.

### 2.3. Diagnostic assessment

**Figure 2.**
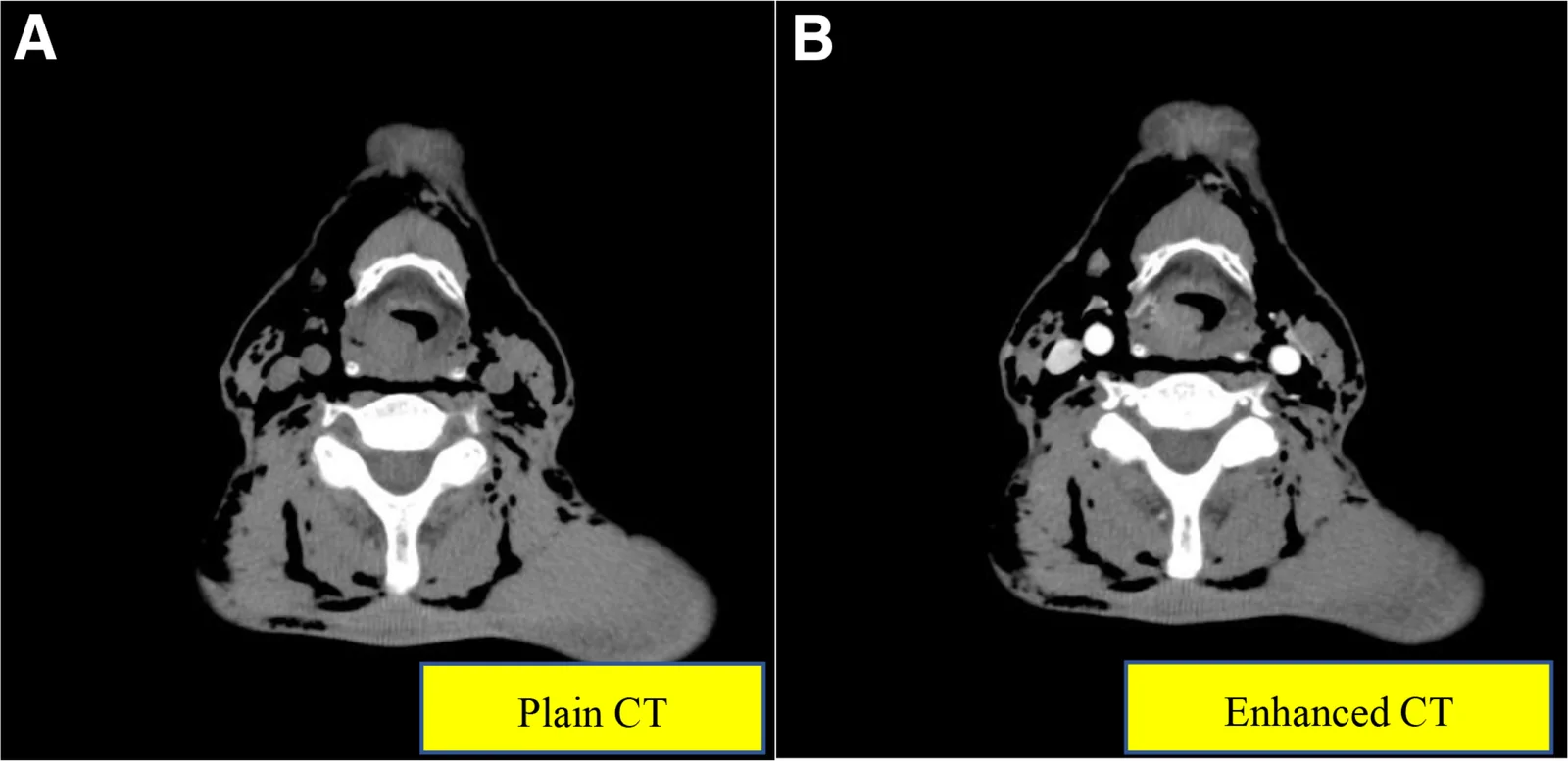
After biopsy, a plain CT scan of the larynx revealed a soft tissue density nodule in the right supraglottic area, with a maximum of 2.1 × 1.9 cm, poorly defined boundaries and uniform density, with an average CT value of about 55 Hu (A). Enhanced CT showed uniform enhancement of the lesions, with an average CT value of about 67 Hu, and thickened branch blood supply of the superior laryngeal artery (B). CT = computed tomography.

Imaging workup: contrast-enhanced neck computed tomography (CT) demonstrated a heterogeneously enhancing lesion in the right supraglottic (2.1 cm × 1.9 cm; Fig. 2); it should be noted that the CT was performed after biopsy-debulking had already reduced tumor bulk.

**Figure 3.**
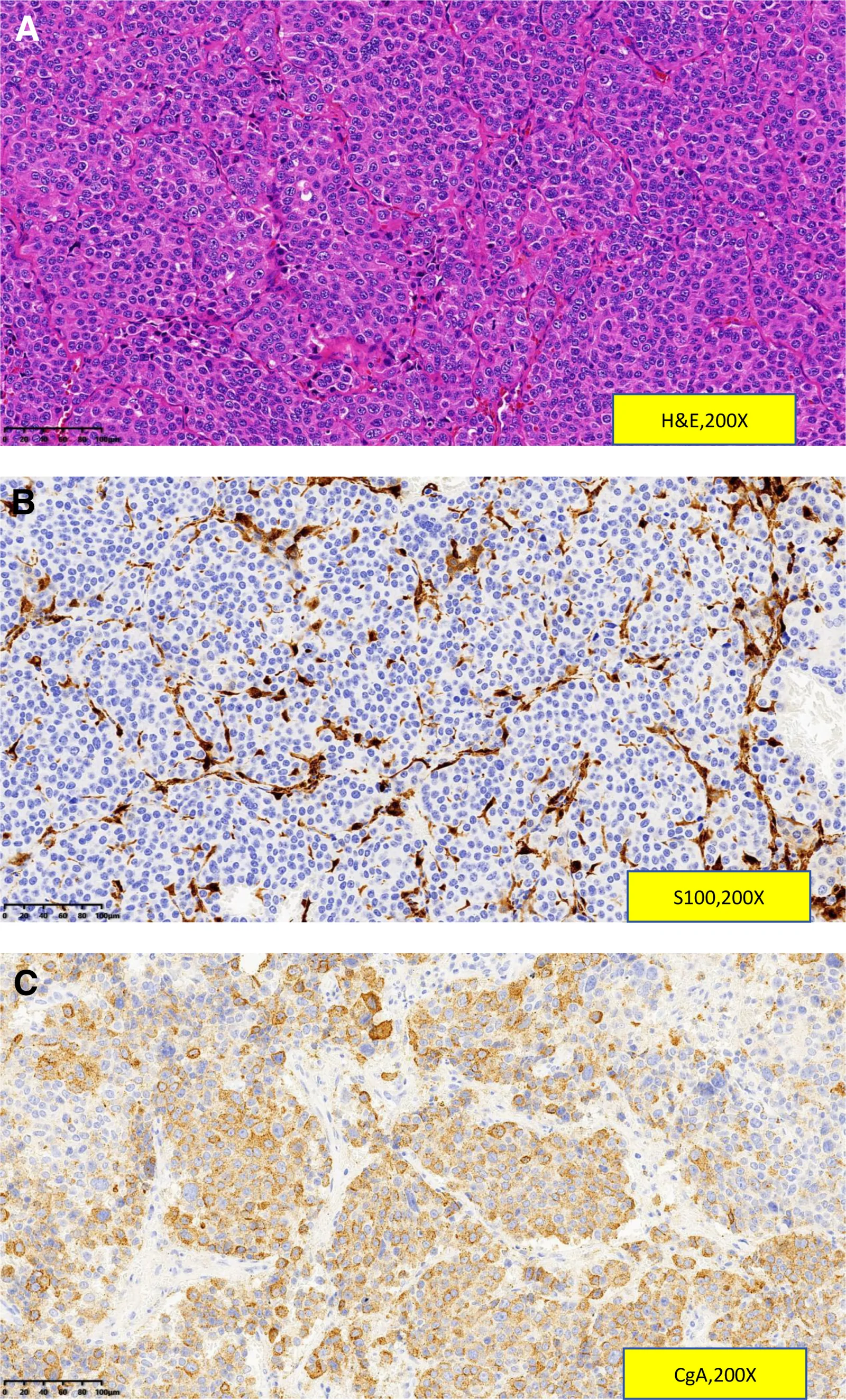
Histopathological and immunohistochemical staining of the primary laryngeal paraganglioma. (A) Hematoxylin and eosin (H&E) staining of biopsy tissue showing epithelioid cells arranged in nests and acini, with large, plasma-rich cytoplasm and rare mitotic figures (scale bar: 100 μm; magnification: 200×). (B) Immunohistochemical staining (EnVision method) showing positive expression of S-100 in sustentacular cells surrounding the cell nests (scale bar: 100 μm; magnification: 200×). (C) Immunohistochemical staining (EnVision method) showing diffuse strong positive expression of chromogranin A (CgA) in tumor cells (scale bar: 100 μm; magnification: 200×).

Histopathology and immunohistochemistry: biopsy revealed the characteristic nested Zellballen architecture with moderate nuclear pleomorphism (mitotic count, 2/10 high-power field). Immunohistochemistry revealed positive results for Syn, CgA, CD56, and S-100, and a Ki-67 index of 10% (Fig. 3, 200×).

### 2.4. Therapeutic intervention timeline

**Figure 4.**
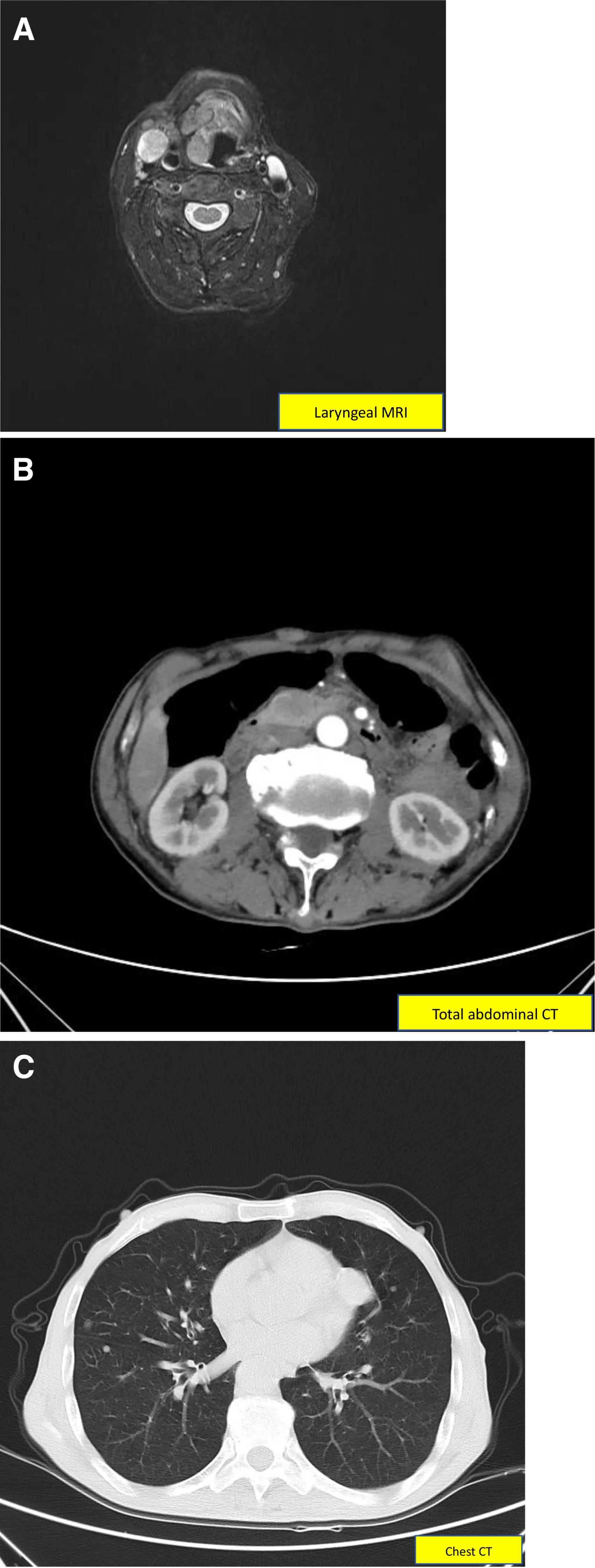
After partial laryngectomy: laryngeal MRI (T2WI) showed multiple slightly high signal nodules in the right supraglottic area and the right neck. Recurrence of paraganglioma with right cervical lymph node metastasis was considered (A). Total abdominal CT enhancement showed a pancreatic head occupying about 2.2 cm in diameter, with unclear borders, and metastasis was considered (B). Chest CT scan showed multiple metastatic tumors in both lungs (C). MRI = magnetic resonance imaging, T2WI = T2-weighted imaging.

Emergency intervention (October 2019) included tracheostomy under local anesthesia (Shiley 7.5), followed by tumor biopsy and debulking under general anesthesia with laryngoscopic support, with an estimated blood loss of 50 mL. The postoperative period was complicated by widespread subcutaneous emphysema in the neck. Definitive surgical management (March 2020) consisted of vertical partial laryngectomy, preserving the left vocal fold; R0 margins were confirmed. Between November 2020 and February 2021, the patient developed biopsy-confirmed subcutaneous metastases on the left chest wall (Syn+, CgA+, CD56+, S-100+, Ki-67 30%). Imaging (enhanced CT/magnetic resonance imaging) revealed local recurrence in the larynx and distant metastases in the pancreatic head, lungs, and para-iliopsoas muscles (Fig. 4). A multidisciplinary team discussion was held. Given the neuroendocrine profile (Syn+, CgA+) of the metastases, peptide receptor radionuclide therapy (PRRT) was considered as a potential option. However, due to the patient’s rapidly declining performance status and explicit wish to forgo further aggressive treatment, PRRT was not initiated, and the patient was discharged with palliative care support (Table 1).

**Table 1:** Timeline.

Date	Event
October 12, 2019, AM	First hospitalization for treatment
October 12, 2019, PM	Emergency tracheostomy + biopsy of laryngeal mass under suspension laryngoscope
October 27, 2019	Discharged against medical advice
March 2, 2020	Readmitted and underwent partial laryngectomy + tumor resection
July 3, 2020	Tracheostomy closure performed
November 22, 2020	Hospital readmission; local tumor recurrence and distant metastasis detected
February 9, 2021	Refused further treatment and died at home after discharge

### 2.5. Follow-up and outcome

The patient developed progressive local recurrence and systemic multisystem metastases (pancreas, lung, para-iliopsoas muscles, etc.) that were detected 13 months after initial diagnosis (November 22, 2020), and subsequently passed away due to multi-organ failure on February 9, 2021.

## 3. Discussion

### 3.1. Epidemiological characteristics and case specificity

This case of primary LP with multisystem metastases provides valuable insight into a rare and aggressive clinical variant. Since its initial description by Blanchard and Saunders,^[5]^ LPs have been typically reported in middle-aged women.^[2,3]^ However, our case involved a 72-year-old male patient, highlighting an atypical epidemiological profile. Several possible explanations exist for this discrepancy. One possibility is the involvement of *SDHx* gene mutations (particularly *SDHB*/*SDHD*) in modulating tumorigenic pathways, which warrants comprehensive germline genetic analysis in future cases. Alternatively, this case may represent a sporadic aggressive variant driven by other molecular mechanisms, such as a high Ki-67 proliferation index alone or additional unidentified genetic alterations. It is also possible that the aggressive behavior reflects a combination of multiple factors rather than a single genetic cause. In this case, *SDHB* immunostaining and germline genetic testing for *SDHx* mutations (e.g., *SDHB*/*SDHD*) were not performed, not due to medical negligence but because clinical priorities focused on life-threatening conditions, the patient’s treatment preferences, and the rapid disease progression. Therefore, while *SDHx* mutation remains a plausible hypothesis, it should be considered alongside other potential mechanisms. This case report emphasizes the importance of considering genetic counseling and testing in future atypical or aggressive LP presentations while also recognizing that the underlying etiology may be multifactorial.

### 3.2. Anatomic localization and molecular pathogenesis

Over 85% of LPs originate from the superior laryngeal paraganglia,^[3]^ anatomically associated with the superior laryngeal nerve and vessels. Intraoperative mapping combined with contrast-enhanced CT confirmed the right supraglottic origin, consistent with classic topography, notably the nonfunctional nature of this tumor with no catecholamine-related symptoms and normal plasma-free metanephrines, despite strong immunohistochemical expression of Syn(+) and CgA(+).

### 3.3. Diagnostic challenges and technological advancements

#### 3.3.1. Diagnosis relied on 2 critical aspects

Histopathological benchmark: the characteristic Zellballen architecture with sustentacular S-100 positivity remains essential for differentiating LP from schwannomas (diffuse S-100+) and carcinoids (cytokeratin+).

Metastatic criteria: multisystem dissemination (subcutaneous, pancreatic, and pulmonary) was validated by contrast-enhanced CT, magnetic resonance imaging, and post-biopsy immunohistochemistry (Syn+, CgA+, CD56+, S-100+, and Ki-67 [30%]), fulfilling the World Health Organization criteria for metastatic disease.

### 3.4. Therapeutic paradigms and prognostic implications

Although LP generally exhibits a favorable prognosis (5-year survival > 90%), this case demonstrated aggressive behavior, with local recurrence and distant metastases detected 13 months after initial diagnosis (November 22, 2020), and the patient died on February 9, 2021. Notably, the patient initially refused partial laryngectomy, leading to an approximately 5-month delay between the initial diagnostic debulking and definitive surgical resection. This interval likely contributed to the rapid disease progression observed, emphasizing that “early diagnosis” in the abstract’s lessons must be coupled with timely definitive surgical intervention to alter the clinical trajectory.

#### 3.4.1. Key management insights

Surgical precision: complete resection with clear margins (>2 mm) (R0) is paramount, as delays in definitive surgery – exemplified by this case – may precipitate rapid recurrence.

Multidisciplinary integration: metastatic LP demands coordinated management with PRRT, hypoxia-inducible factor-2 alpha inhibitors (Belzutifan), and stereotactic radiosurgery.

Risk stratification: proposed prognostic markers include *SDHB* loss (≥10% tumor cells), Ki-67 ≥5%, and tumor volume >3 cm.

## 4. Conclusion

This report provides a detailed, longitudinal account of an aggressive LP case, emphasizing diagnostic, therapeutic, and prognostic considerations. Limitations include the absence of *SDHx* genetic testing and the single-patient observational design, limiting generalizability. Primary LP can exhibit aggressive behavior with rapid multisystem metastases, particularly in atypical patient populations. Early recognition, complete surgical resection, and multidisciplinary management are essential to optimize outcomes. Targeted therapy with hypoxia-inducible factor-2 alpha inhibitors (e.g., Belzutifan) may be considered in selected cases with evidence of von-Hippel-Lindau/hypoxia-inducible factor pathway activation, although its use in sporadic metastatic paraganglioma remains investigational.^[6,7]^ Genetic counseling should be considered for atypical or aggressive presentations.

## Author contributions

**Visualization:** Xia Xu.

**Resources:** Shuting Ma.

**Software:** Shuting Ma.

**Funding acquisition:** Yaping Zou.

**Writing – original draft:** Xia Xu, Yaping Zou.
